# Improvement over time in outcomes for patients undergoing endoscopic therapy for Barrett's oesophagus-related neoplasia: 6-year experience from the first 500 patients treated in the UK patient registry

**DOI:** 10.1136/gutjnl-2014-308501

**Published:** 2014-12-24

**Authors:** R J Haidry, M A Butt, J M Dunn, A Gupta, G Lipman, H L Smart, P Bhandari, L Smith, R Willert, G Fullarton, M Di Pietro, C Gordon, I Penman, H Barr, P Patel, N Kapoor, J Hoare, R Narayanasamy, Y Ang, A Veitch, K Ragunath, M Novelli, L B Lovat

**Affiliations:** 1 Research Department of Tissue and Energy, Division of Surgery and Interventional Science, University College London, London, UK; 2 Department of Gastroenterology, University College Hospital NHS Foundation Trust, London, UK; 3 Guy's and St Thomas’ NHS foundation Trust, London, UK; 4 Institute for Cancer Genetics and Informatics, Oslo University, Oslo, Norway; 5 Department of Gastroenterology and Hepatology, Royal Liverpool University Hospital, Liverpool, UK; 6 Princess Alexandra Hospital, Portsmouth, UK; 7 Bradford Teaching Hospitals NHS Foundation Trust, Bradford, UK; 8 Central Manchester University Hospitals NHS Foundation Trust, Manchester,UK; 9 Glasgow Royal Infirmary, Glasgow, UK; 10 Addenbrooke's Hospital, Cambridge, UK; 11 Royal Bournemouth Hospital, Bournemouth, UK; 12 Royal Infirmary Edinburgh, Edinburgh, UK; 13 Oesophagogastric Surgery, Gloucestershire Hospital NHS Trust, Birmingham, UK; 14 Department of Gastroenterology, Southampton University Hospital, Southampton, UK; 15 Digestive Diseases Centre, Aintree University Hospital, Liverpool, UK; 16 St Mary's Hospital NHS Trust, London, UK; 17 St James Hospital, Dublin, Republic of Ireland; 18 Centre of Gastrointestinal Sciences, University of Manchester, Salford Royal Foundation NHS Trust, Salford, UK; 19 Department of Gastroenterology, Royal Wolverhampton Hospitals NHS Trust, Wolverhampton, UK; 20 Department of Gastroenterology, Nottingham University Hospital NHS Trust, Nottingham, UK

**Keywords:** BARRETT'S OESOPHAGUS, OESOPHAGEAL CANCER, ENDOSCOPIC PROCEDURES

## Abstract

**Background:**

Barrett's oesophagus (BE) is a pre-malignant condition leading to oesophageal adenocarcinoma (OAC). Treatment of neoplasia at an early stage is desirable. Combined endoscopic mucosal resection (EMR) followed by radiofrequency ablation (RFA) is an alternative to surgery for patients with BE-related neoplasia.

**Methods:**

We examined prospective data from the UK registry of patients undergoing RFA/EMR for BE-related neoplasia from 2008 to 2013. Before RFA, visible lesions were removed by EMR. Thereafter, patients had RFA 3-monthly until all BE was ablated or cancer developed (endpoints). End of treatment biopsies were recommended at around 12 months from first RFA treatment or when endpoints were reached. Outcomes for clearance of dysplasia (CR-D) and BE (CR-IM) at end of treatment were assessed over two time periods (2008–2010 and 2011–2013). Durability of successful treatment and progression to OAC were also evaluated.

**Results:**

508 patients have completed treatment. CR-D and CR-IM improved significantly between the former and later time periods, from 77% and 56% to 92% and 83%, respectively (p<0.0001). EMR for visible lesions prior to RFA increased from 48% to 60% (p=0.013). Rescue EMR after RFA decreased from 13% to 2% (p<0.0001). Progression to OAC at 12 months is not significantly different (3.6% vs 2.1%, p=0.51).

**Conclusions:**

Clinical outcomes for BE neoplasia have improved significantly over the past 6 years with improved lesion recognition and aggressive resection of visible lesions before RFA. Despite advances in technique, the rate of cancer progression remains 2–4% at 1 year in these high-risk patients.

**Trial registration number:**

ISRCTN93069556.

**Significance of this study**

**What is already known on this subject?**

- High-grade dysplasia and intramucosal cancer arising in Barrett's oesophagus (BE) can carry a 40–60% risk of progressing to oesophageal adenocarcinoma.
- The British Society of Gastroenterology has recently released guidelines recommending that patients with BE-related neoplasia and disease confined to the mucosa (T1a) should be offered endoscopic therapy as first-line treatment.
- The radiofrequency ablation (RFA) registry was founded in 2008 to audit and monitor the outcomes of those undergoing minimally invasive endoscopic therapy.

**What are the new findings?**

- Between 2011 and 2013 there has been a significant improvement in clinical outcomes for patients undergoing endoscopic treatment for BE-related neoplasia.
- Reversal of all dysplasia has risen from 77% to 92% at 12 months compared with patients treated between 2007 and 2010.
- Between 2011 and 2013 the progression to invasive cancer at 12 months was 2.1% and the calculated cancer risk at almost 34 months is 3%.
- Latterly, endoscopic mucosal resection is more widely used prior to initiating RFA, but the risk of symptomatic stenosis requiring endoscopic therapy has not changed over time.

**How might it impact on clinical practice in the foreseeable future?**

- There is now consensus that first-line treatment for mucosal neoplasia arising in BE should be endoscopic therapy.
- Lesion recognition and resection prior to RFA are paramount to successful outcomes in patients with BE neoplasia. Visible and nodular lesions are more likely to harbour more advanced neoplasia, so early resection is key to both definitive staging and eradication prior to RFA.

## Introduction

Patients with Barrett's oesophagus (BE) are at increased risk of developing oesophageal adenocarcinoma (OAC). BE arises in response to chronic acid reflux and has a population prevalence in the region of 1.6%.^1^ The incidence of OAC continues to rise in the Western world.^2–4^ Despite advances in medical care, the prognosis from this disease is poor with a 5-year survival of 7% in England and Wales. Although metaplastic BE confers a low risk of progression to OAC in the region of 0.12–0.40 per year,^5–7^ the presence of dysplasia in BE can increase this risk significantly. High-grade dysplasia (HGD) and intramucosal cancer (IMC) (collectively referred to as BE-related neoplasia) arising in BE are now generally believed to carry a 40–60% risk of progressing to OAC.^8^ The treatment options for patients with BE-related neoplasia have shifted dramatically over the past few years, from radical surgery with oesophagectomy towards minimally invasive endoscopic therapy.

For many years the preferred strategy for managing these patients has been either intensive endoscopic surveillance until cancer develops or surgery with oesophagectomy to cure the disease. Indeed, in patients undergoing surgery for HGD, more advanced disease used to be found in 40% of cases,^9^ although more recently with better endoscopic imaging this has fallen to 17%.^10^ Surgical techniques continue to improve and, although the operative mortality has fallen from as high as 12% in the 1990s,^11–13^ it remains not insignificant at the present time. Data from the UK National Oesophago-Gastric Cancer Audit (NOGCA) in 2012 demonstrated an intraoperative mortality of 2–4% for all patients, although for patients undergoing surgery for HGD the surgical mortality is lower at 1%.^14^ In addition, the subsequent morbidity can be 40%^15^ and it usually takes at least 9 months before patients return to a normal quality of life.^16^ ^17^

Given the morbidity and mortality that may be associated with oesophagectomy, less invasive endoscopic treatment modalities have emerged. The British Society of Gastroenterology (BSG) has recently released guidelines recommending that patients with BE-related neoplasia and disease confined to the mucosa (T1a) should be offered endoscopic therapy as first-line treatment. It should be offered only in specialist high volume centres where the expertise and support is in place to carry out these interventions.^18^

The rationale for endoscopic therapy is that the risk of distant metastasis is low.^19^ ^20^ Once there is submucosal involvement (T1b disease) this risk is greater (up to 20%), and endoscopic therapy should no longer be considered curative.^21^ ^22^ Published data from limited randomised studies^23^ have been followed up by several larger series from prospective studies showing short-term efficacy and encouraging long-term durability while maintaining a good safety profile for endoscopic therapy with radiofrequency ablation (RFA) and endoscopic mucosal resection (EMR). Real-life data demonstrating the effectiveness of these therapies in large volume registry populations has again shown sustained promise.^24–26^

The aims of the present study were: (1) to assess the long-term clinical efficacy of RFA/EMR as first-line treatment for over 500 patients with BE-related neoplasia within the UK registry since its inception in 2008; (2) to establish whether, after 6 years of endoscopic therapy, there has been any change in clinical outcomes for these patients by comparing patients treated in the first 3 years of the registry with those treated in the second 3-year period; and (3) to determine factors that may be associated with changes in outcome.

## Methods

### Inclusion criteria

All patients were referred for consideration of endoscopic management of dysplastic BE at a specialist centre within the UK registry. Only men and non-pregnant women over the age of 21 years with no contraindications to endoscopy were considered. All patients gave written informed consent and agreed to attend at regular intervals for treatment and surveillance procedures.

### Pretreatment staging

All patients with a diagnosis of BE neoplasia were discussed within specialist multidisciplinary team (MDT) meetings at designated centres within the UK Upper GI Cancer Network in compliance with BSG guidelines. Those diagnosed at smaller local hospitals were referred on and restaged at the regional specialist centre performing RFA/EMR.

The registry captures data on all patients treated regionally at the designated centres. These centres are selected by each regional cancer network and have the necessary training and support infrastructure to carry out safe and effective endoscopic therapy on any patients with BE neoplasia referred to them. Although the registry protocol describes recommended best practice, there is invariably variation in treatment and follow-up intervals depending on local service limitations. The data are collected through an online anonymised database where demographic variables are captured together with treatment-specific information on type of treatment (RFA/EMR), resection histology, outcomes and adverse events. These data capture real-life outcomes of high quality novel endoscopic therapy offered within the confines of challenging service provision.

At endoscopy the BE segment was mapped and measured using the Prague classification^27^ in centimetres. Enhanced endoscopic imaging techniques were used where available, such as narrow band imaging (Olympus, Hamburg, Germany), i-scan (Pentax, Hoya Corporation, Japan) and flexible spectral imaging colour enhancement (FICE) (Fujinon, Saitama, Japan) to help highlight potential dysplastic lesions for assessment and treatment. All investigators used the Paris classification^28^ to classify visible lesions. Endoscopic ultrasound was performed at the discretion of the operator if visible lesions were identified to exclude T2 disease and mediastinal lymphadenopathy which would preclude endoscopic therapy. The registry protocol mandated that EMR should be carried out for all visible lesions and nodules where identified for therapeutic as well as staging purposes. All nodularity was removed before RFA to the residual flat BE segment. The BE mucosa was sampled using the Seattle 4 quadrant biopsy protocol.^29^ ^30^ If invasive cancer was demonstrated, patients were referred via their regional cancer MDT for consideration of radical or palliative therapy.

A minimum of two endoscopies showing HGD or IMC was mandatory prior to performing RFA. EMR could be carried out at any time. Histology was reviewed by two expert gastrointestinal histopathologists at the individual centres. The modified Vienna classification^31^ was used to categorise pathological findings before treatment decisions were made.

### Registry endoscopy protocol

RFA was commenced with either the circumferential ablation device (HALO 360) or with one of several focal RFA devices for shorter non-circumferential areas of BE (HALO 90, HALO 60, HALO ULTRA, Channel HALO device). The first RFA treatment was deemed the start of the treatment protocol for all patients, even after previous EMR. The treatment protocol is identical to that used in our previous publications and has remained unchanged over the two time periods being analysed here.^24^ The protocol has been designed to mirror the algorithm set out in the first randomised controlled trial on RFA in patients with BE neoplasia with the recommended end of protocol defined as 12 months at which stage patients were said to have completed treatment.^23^ In some patients with very short segments of BE where clearance of disease was achieved sooner than the suggested 12 months, the endpoint was the first endoscopy after the completion of treatment where there was no neoplasia and BE on biopsy.

**Figure 1:**
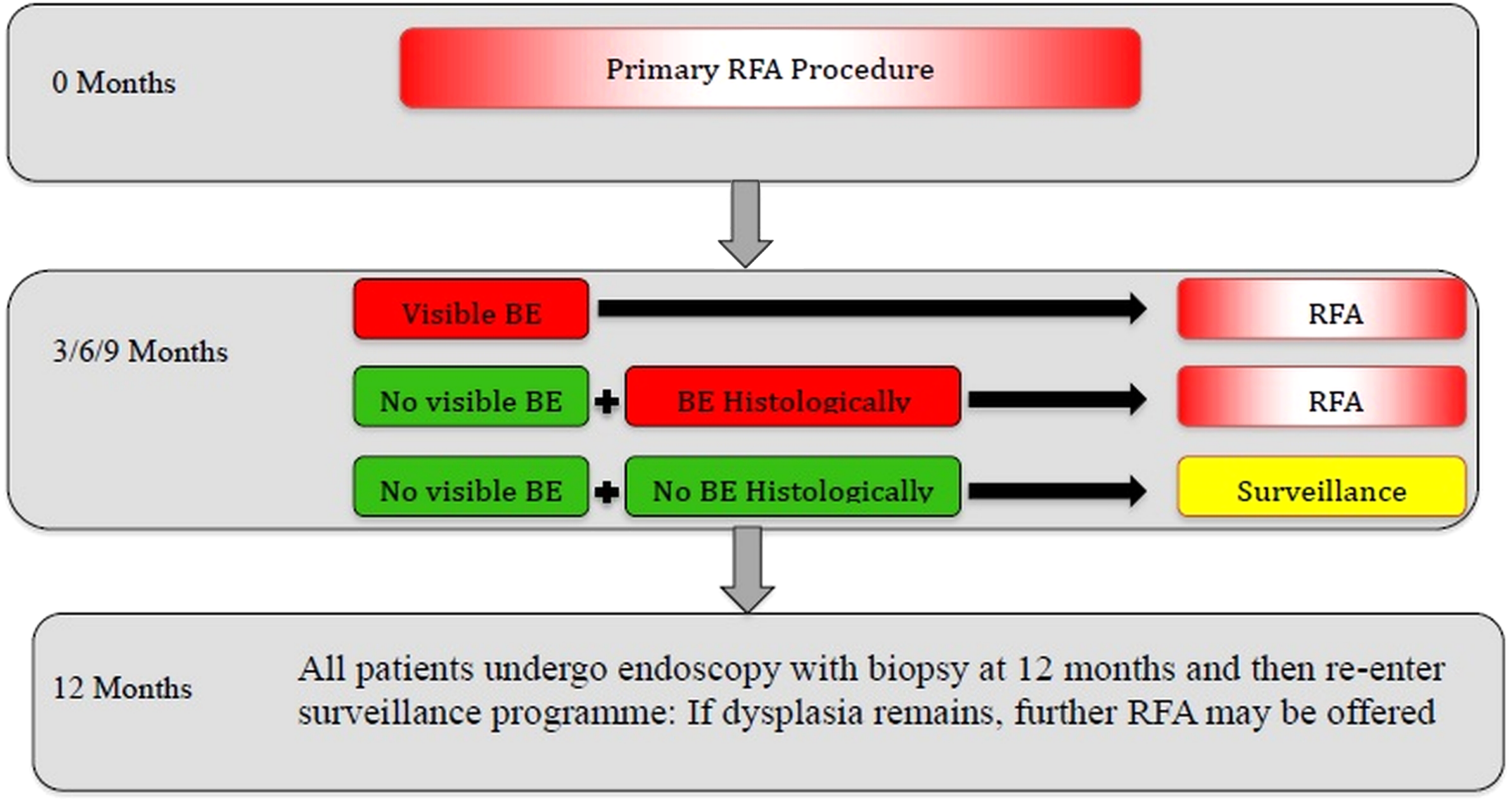
UK radiofrequency ablation (RFA) registry protocol. The treatment protocol started following the first RFA treatment even in patients who had had prior RFA. BE, Barrett's oesophagus.

The mean number of RFA sessions was 2–3 during the treatment protocol in both time periods. All patients were maintained on omeprazole 40 mg twice daily or equivalent during the treatment period. A flow chart of the registry protocol is shown in figure 1.

Patients free of dysplasia or BE at end of protocol were entered into a post-RFA surveillance protocol and were followed up at 3-month intervals for the first year, 6-month intervals for the second year and annually thereafter. Biopsies were taken from 1 cm below the neo z-line and from the previously treated BE segment using the Seattle protocol in line with the BADCAT recommendation.^32^ Endoscopy with enhanced imaging was advocated in all these cases to look for recurrences.

In cases where there was residual dysplasia at the end of the suggested treatment protocol, further endoscopic therapy was offered with either RFA or EMR depending on the morphology of the remaining neoplasia at endoscopy.

If biopsy-proven recurrence of dysplasia occurred after initial successful therapy and was amenable to further RFA/EMR, this was offered to the patient. Recurrence of intestinal metaplasia (IM) after successful endoscopic eradication was judged to be a true recurrence only when there was histological confirmation of IM proximal to the neo-oesophagogastric junction (OGJ) (>5 mm) in the previously treated BE segment. This mirrors similar long-term follow-up studies where recurrence was defined as IM in biopsies 3–10 mm above the neo-squamocolumnar junction (SCJ) at the gastro-oesophageal junction (GOJ).^33^ ^34^

Following RFA, the biopsy protocol recommended samples were taken above and below the neo-OGJ. Focal IM distal to the neo-OGJ and from the gastric cardia was not deemed to be true recurrence or criteria for retreatment. The presence of IM in the gastric cardia or at the GOJ is a common pathological finding at endoscopy and can occur in 5–18% of the normal population.^35^ ^36^ There is evidence that individuals with IM at the cardia or GOJ have a significantly lower cancer risk than patients with BE and therefore, if this was found on follow-up biopsy, it was not re-treated.^37^

If the patients progressed to invasive disease at any time and were no longer curable with endoscopic therapy, they were referred back to the regional specialist MDT to discuss eligibility for surgery or chemoradiotherapy. For patients who progressed to invasive disease within the 12-month protocol period, data were censored at their last procedure.

### Clinical endpoints

The primary outcomes for the registry are clearance rate (CR) for all dysplasia (CR-D), which was defined as no biopsy-proven residual dysplasia of any grade in the previously treated Barrett's segment. Clearance of all BE (CR-IM) was defined by no evidence of IM on biopsy at end of protocol from their index RFA treatment. In addition, progression to invasive cancer and overall cancer risk were observed in all patients in the registry. Long-term outcomes and durability for reversal of dysplasia for those with a favourable outcome at end of protocol were also assessed. Clinical outcomes, patient demographics and treatment trends were then analysed and compared between those who started treatment in 2008–2010 and those enrolled in 2011–2013.

### Statistical considerations

Quantitative endpoints such as clearance of dysplasia at 12 months were compared with the patient's baseline status using Cox proportion hazard models, Student t tests and Kaplan–Meier (KM) survival curves. Comparisons of outcome measures between the two time periods were analysed with χ^2^ tests. Binomial logistic regression was performed on the entire dataset and for each separate time period to assess the influence of measured variables on the likelihood of achieving CR-D.

## Results

### Registry enrollment and demographic data

Between 2008 and 2013 a total of 920 patients were treated in the UK registry at 25 centres nationwide. Of these, 508 have now completed treatment (266 patients in 2008–2010 and 242 patients in 2011–2013). Demographic data are shown in table 1. There was no significant difference between the two time periods with regard to patient age, number of RFA treatment sessions or baseline length of BE being treated. Baseline histology was not significantly different in both groups between the two time periods. The majority of patients (overall 73%) were treated for HGD with the remainder being treated for IMC, apart from 3% who were treated for low-grade dysplasia. This has not changed over the 6-year period.

**Table 1:** Comparison of demographic and pre-RFA characteristics for all patients undergoing endoscopic therapy for BE-related neoplasia between 2008–2010 and 2011–2013

	2008–2010	2011–2013	p Value
No of patients completing treatment protocol	266	242	NS
Mean age (range)	68 (40–87)	69 (44–90)	NS
M:F (%)	80:20	84:16	NS
Baseline histology, n (%)			NS
LGD	7 (3%)	7 (3%)	
HGD	197 (74%)	172 (71%)	
IMC	62 (23%)	63 (26%)	
Previous photodynamic therapy (%)	9	3	0.02
Baseline BE length at start of RFA (maximum extent), cm	6 (1–20)	4.7 (1–16)	NS
EMR prior to RFA, n (%)	128 (48%)	143 (60%)	0.016
Rescue EMR during RFA treatment, n (%)	35 (13%)	4 (2%)	<0.0001
Mean time to end of protocol (months)	12.6	10.3	NS
Mean no of RFA treatments during treatment protocol	2.6 (1–5)	2.5 (1–5)	NS

BE, Barrett's oesophagus; EMR, endoscopic mucosal resection; HGD, high-grade dysplasia; IMC, intramucosal cancer; LGD, low-grade dysplasia; NS, not statistically significant; RFA, radiofrequency ablation.

Thirty-eight per cent of patients were treated in the two largest centres while the remaining cases were treated between the other 23 centres. In 2008–2010, 17 centres nationwide were providing endoscopic therapy for BE-related neoplasia, contributing outcomes to the registry. This had risen to 25 by 2013.

### Patient outcomes 2008–2010

**Figure 2:**
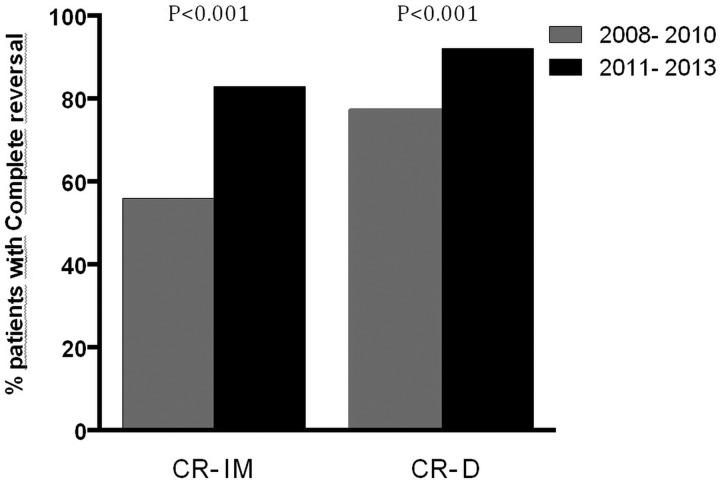
Graph showing improvement in complete reversal of intestinal metaplasia (CR-IM) and complete reversal of dysplasia (CR-D) in patients undergoing radiofrequency ablation/endoscopic mucosal resection between 2008–2010 and 2011–2013.

Between 2008 and 2010, 266 patients were treated and completed the treatment protocol. During this time, 12-month CR-D was achieved in 77% of patients and reversal of IM (CR-IM) in 57% (table 2). Of these patients, 48% required EMR for visible lesions before they underwent RFA and a further 13% needed rescue EMR after the start of RFA treatment for new lesions. Nine patients (3.6%) progressed to invasive cancer by 12 months and a total of 18 (6.7%) of these patients have developed invasive cancer at most recent follow-up (median follow-up 31 months from end of treatment; range 3–72 months). KM survival analysis shows that at 5 years the estimated risk of progression to cancer was 11% in this cohort (figure 2).

**Table 2:** Comparison of end of protocol treatment and long-term clinical outcomes for all patients undergoing endoscopic therapy for BE-related neoplasia between 2008–2010 and 2011–2013

	2008–2010	2011–2013	p Value
CR-IM at end of protocol in all patients	152/266 (57%)	201/242 (83%)	<0.0001
CR-IM in patients with HGD	109/197 (55%)	145/172 (85%)	
CR-IM in patients with IMC	38/62 (61%)	51/63 (81%)	
CR-IM in patients with LGD	5/7 (71%)	5/7 (71%)	
CR-D at end of protocol in all patients	206/266 (77%)	222/242 (92%)	<0.0001
CR-D in patients with HGD	149/197 (76%)	159/172 (92%)	
CR-D in patients with IMC	50/62 (81%)	57/62 (92%)	
CR-D in patients with LGD	7/7 (100%)	6/7 (86%)	
Progression to cancer at end of protocol	9/266 (3.4%)	5/242 (2.1%)	0.51
Progression to cancer at most recent follow-up	18/266 (6.7%)	6/242 (2.5%)	Log rank 0.085
Median time to most recent biopsy from first treatment for those still in follow-up (months)	31 (3–72) n=218	13 (2–32) n=211	
% free of dysplasia at most recent follow-up	97%	96%	Log rank 0.2
% free of IM at most recent follow-up	91%	94%	Log rank 0.02
Symptomatic stricture requiring endoscopic dilation	25/266 (9.4%)	15/242 (6.2%)	0.18

CR-D, complete reversal of dysplasia; CR-IM, complete reversal of intestinal metaplasia; HGD, high-grade dysplasia; IM, intestinal metaplasia; IMC, intramucosal cancer; LGD, low-grade dysplasia; NS, not statistically significant.

The logistic regression model identified that increasing age (OR 1.04, 95% CI 1.004 to 1.078, p=0.03) and shorter lengths of BE at baseline (OR 1.099, 95% CI 1.003 to 1.203, p=0.04) were marginally more likely to achieve CR-D during this initial time period. Conversely, rescue EMR during the treatment protocol was associated with a reduction in the likelihood of achieving CR-D in the initial treatment period (OR 0.4, 95% CI 0.176 to 0.907, p=0.028). Prior EMR and time to end of protocol did not influence the outcomes.

### Patient outcomes 2011–2013

**Figure 3:**
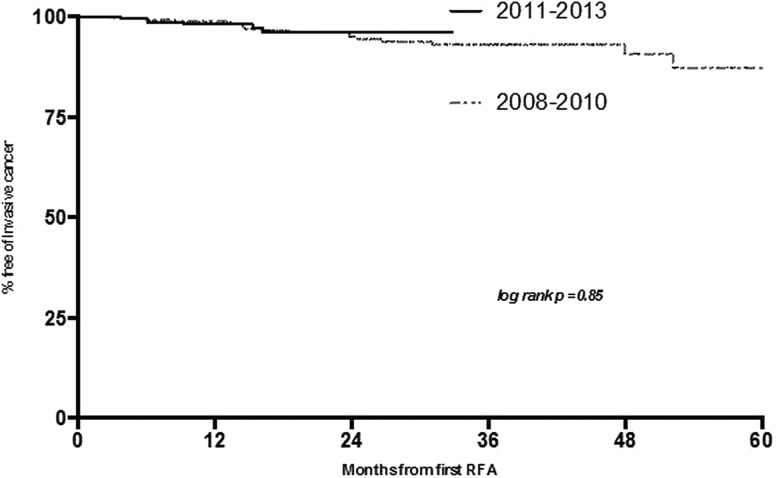
Kaplan–Meier analysis demonstrating estimated cancer progression from start of treatment in patients undergoing endoscopic therapy for Barrett's oesophagus-related neoplasia over the two time periods.

In the time period 2011–2013 a further 242 patients were treated and 12-month biopsy results were available. There was a significant improvement in outcomes with 92% CR-D and 83% CR-IM (p<0.0001) compared with patients treated in 2007–2010 (figure 3). Furthermore, the use of EMR prior to RFA increased to 60% (p=0.016). Conversely, the requirement for rescue EMR after the start of RFA treatment was reduced from 13% to only 2% (p<0.0001). In 2011–2013 the progression to invasive cancer at 12 months was 2.1% and this did not change (p=0.51). For this group, the KM calculated cancer risk at almost 34 months was 3% (figure 3). Taking into consideration the fact that the follow-up periods in these two groups were different, there is currently no overall statistical significance in long-term cancer progression (log rank p=0.85).

The logistic regression model found that the previously significant influence of age, initial length of BE and rescue EMR on achieving CR-D did not remain in this period. No other variables were found to influence outcomes significantly.

When the logistic regression model was applied to the entire dataset for 2008–2013, increasing age (OR 1.316, 95% CI 0.685 to 2.529), prior EMR (OR 1.358, 95% CI 0.776 to 2.337) and shorter lengths of BE at baseline (OR 1.103, 95% CI 1.023 to 1.190) were more likely to achieve CR-D. Again, rescue EMR (OR 0.426, 95% CI 0.195 to 0.930) reduced the chances of successful treatment.

Despite the increased use of EMR latterly, the risk of symptomatic stenosis requiring endoscopic therapy did not change significantly over time (9.4% in 2007–2010 vs 6.2% in 2011–2013, p=0.18; table 2).

There was a single perforation in the very early experience of the study with a 34 mm ablation balloon that is now no longer available. The patient made a full recovery and went on to achieve disease clearance. The rate of bleeding was <1% in both time periods, none of which required blood transfusion.

### Durability of response

**Figure 4:**
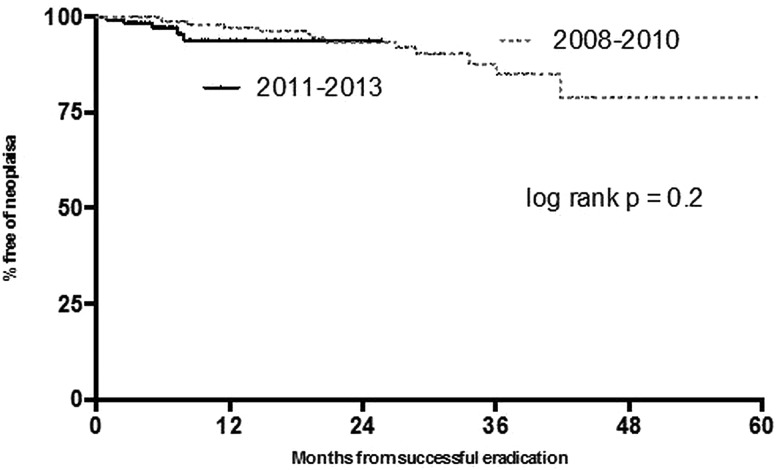
Kaplan–Meier survival statistics showing durability of neoplasia reversal in patients treated within the two time periods.

Long-term durability of neoplasia reversal was assessed in these two cohorts using KM survival statistics. This demonstrated that, in the earlier time period, the risk of neoplasia recurrence was in the region of 19% at 5 years. Taking into account the shorter follow-up period in patients treated more recently, the risk of recurrence at 26 months was 7%. These data are not statistically significant (log rank p=0.2, figure 4).

**Figure 5:**
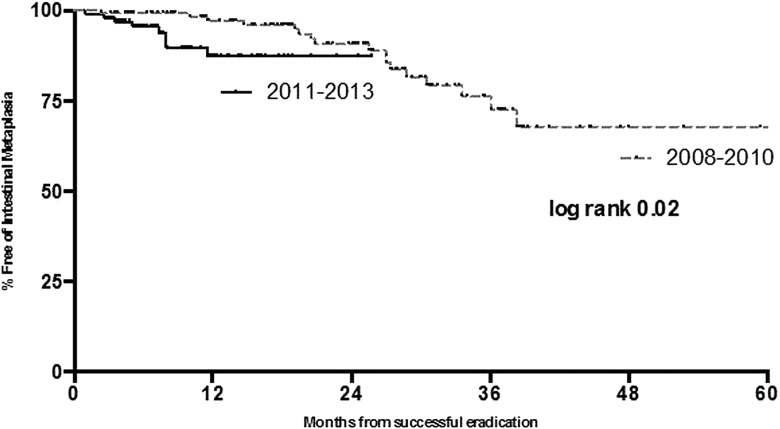
Kaplan–Meier survival statistics showing durability of intestinal metaplasia reversal in patients treated within the two time periods.

With respect to reversal of IM over the two time periods, the predicted risk of IM recurrence was 13% at 26 months in patients treated in the most recent time period. At the same time period the predicted IM recurrence was 12% in patients treated between 2008 and 2010. At 5 years there is a 32% risk of IM recurrence in this cohort of patients. It appears from KM statistics (figure 5) that the majority of IM recurrences in patients treated more recently tend to occur within the first 12 months after treatment, following which this risk plateaus.

### Outcomes of cancer patients

A total of 24 patients progressed to invasive disease that was no longer amenable to endoscopic therapy. Ten of these 24 patients were treated with oesophagectomy, one of whom died intraoperatively. Two patients presented with metastatic disease; one was lost to follow-up and the remaining 11 patients had locally advanced disease that was treated with palliative therapy ranging from chemoradiotherapy or endoscopic laser therapy for obstructive symptoms.

## Discussion

There is now consensus that first-line treatment for mucosal neoplasia arising in BE should be endoscopic therapy.^18^ Following the first randomised controlled trial demonstrating efficacy and safety of RFA in carefully selected patients with low-grade dysplasia (LGD) and HGD,^23^ there have been numerous further large-volume multicentre prospective series demonstrating similar rates of efficacy worldwide.^24^ ^25^ ^34^ ^38^ Surgical outcomes for those undergoing oesophagectomy have also improved and, for those with more advanced neoplasia infiltrating the submucosa and beyond, this should be considered if there is no distant spread. Recent guidelines from the BSG endorse this management.^18^

Our data demonstrate that clinical outcomes for patients with mucosal BE neoplasia undergoing endoscopic therapy have improved since 2008. Furthermore, the practice of EMR being more widely used prior to initiating RFA may explain the improved outcomes. The manner in which the registry data are collated does not capture the extent of EMR before RFA and whether this changed over time. One could argue that, if larger areas were resected at baseline in the later time period, this would leave less residual BE which would require fewer RFA treatment sessions. The higher proportion of patients undergoing EMR may therefore account for the observed difference, although the number of RFA sessions did not actually fall in the second time period.

An alternative explanation from our data is that EMR eradicated the visible and nodular neoplasia, making treatment more successful in the latter time period. In our opinion, lesion recognition and resection are paramount to successful outcomes in patients with BE neoplasia. Visible and nodular lesions are more likely to harbour more advanced neoplasia, so early resection is key to both definitive staging and eradication prior to possible RFA. However, despite the known efficacy of combined RFA and EMR for these patients, treatment can be limited by the ability to find the areas that need to be treated. The accurate detection of neoplasia is an important objective that incorporates both new technologies in endoscopic imaging and continuing understanding of recognising abnormalities of the mucosa and microvasculature by the endoscopist.

Endoscopic imaging has dramatically improved over the past 5 years, allowing endoscopists to assess mucosal anomalies in detail and enabling accurate sampling of suspected areas of neoplasia and direct therapy. White light endoscopy alone, with random sampling of the tubular oesosphagus, can often overlook lesions that require resection. High-resolution video endoscopes with high-quality charge-coupled device chips (up to 1.2 million pixels) allow inspection of minute details of the mucosal and vascular architecture that were not readily visible with previously used white light endoscopes. High-definition endoscopy with various imaging enhancements is now practised at most high-volume centres performing therapy for BE neoplasia. There are increasing data to support improved diagnostic yield with enhanced imaging. In 2012 Sharma *et al* compared narrow band imaging (NBI) with standard high-definition white light endoscopy (HD-WLE) to detect IM and neoplasia in patients undergoing BE assessment and found that neoplasia detection was higher with NBI than with HD-WLE (30% vs 21%, p=0.01).^39^ Pohl *et al*^40^ demonstrated that, by targeting biopsies with HD-WLE and image enhancement, the sensitivity and specificity rates for BE neoplasia were 96.7% and 66.5%, respectively.

The use of chromoendoscopy as an adjunct to enhanced imaging technology and high-definition endoscopy has become more widespread and allows more accurate diagnosis of neoplasia. Several studies have shown that the use of acetic acid in patients with BE can improve neoplasia yield.^41^

Improved lesion recognition with advancements in endoscopic technology and training of endoscopists has led to more aggressive and widespread use of EMR prior to RFA. The more widespread use of EMR in the past 3 years of the registry has, in our opinion, led to improved clinical outcomes and can be seen to provide two potential benefits. First, it serves as a larger and deeper biopsy specimen, allowing more precise determination of the depth of tumour penetration than any other method currently available. Most specimens contain significant portions of the submucosa, allowing differentiation of mucosal from submucosal tumours. EMR has been shown to have improved sensitivity over endoscopic ultrasound for differentiating submucosal involvement in patients with BE neoplasia.^42^ Submucosal involvement in patients with visible lesions carries a significant risk of lymph node metastasis at 5 years. Those with neoplasia confined to the superficial 500 μm of the submucosa (T1b, SM1 disease) still have a rate of lymph node metastases of 1–4% at 5 years.^43^ With increased use of EMR before RFA, patients with submucosal disease can be filtered out and offered surgery rather than further endotherapy with curative intent as they are less likely to have a favourable outcome. Second, it can be performed with curative intent for visible lesions allowing for RFA of the residual flat BE segment. Indeed, EMR has been shown to be very effective alone in the treatment of BE-related neoplasia^44^ as well as when combined with RFA. Within the registry the increased use of EMR prior to RFA in the past 3 years has seen a dramatic fall in the need for rescue EMR. One could postulate that the limited use of EMR in the first 3 years of the registry experience meant that more patients needed rescue EMR, and this corresponded to the less impressive clinical outcomes.

While improved endoscopic imaging and more EMR could explain the improved outcomes, other parameters may have also contributed. These include increased physician awareness, improved disease staging, better patient selection and improved endoscopic skills in this novel area of therapeutics. Over the next few years it is conceivable that long-term outcomes for these high-risk patients may improve further.

Recent publications have explored predictors of successful RFA treatment and predictors of disease recurrence. Van Vilsteren *et al*^45^ identified four predictive factors for a poor initial response after circumferential RFA at 3 months. These were active reflux oesophagitis, regeneration of BE over the site of a previous endoscopic resection, pre-existing oesophageal narrowing before RFA and the number of years of neoplasia pre-RFA. Pasricha *et al* showed from the US RFA registry that more advanced pretreatment histology was associated with an increased recurrence rate. They also demonstrated that patients with recurrence were more likely to be older, have longer BE segments, be non-Caucasian, have dysplastic BE before treatment and require more treatment sessions.^26^

We have shown in our previous data that CR-D was 15% less likely for every 1 cm increment in BE length (OR 1.156; SE 0.048; 95% CI 1.07 to 1.26; p<0.001).^24^ In our current series the BE length was marginally shorter in the later time period (4.7 cm in 2011–2013 vs 6.0 cm in 2008–2010) and may also therefore have been a contributing factor to the improved disease reversal rates observed.

Overall progression to invasive disease in patients with BE is quoted to be in the region of 0.12–0.40% per annum.^5–7^ In our registry data the rate of progression in all patients undergoing endoscopic therapy for BE-related neoplasia fell to 2.1% at 12 months in the past 3 years compared with 3.4% in the initial time period. The durability of cancer-free survival between the two groups does not appear to be significantly different. However, the follow-up periods are different. These patients are biologically at high risk despite endoscopic intervention. It remains to be seen whether the better reversal rates of dysplasia translate into a long-term reduction in cancer incidence.

Combined RFA and EMR for carefully selected patients with BE neoplasia is now an established alternative to more invasive surgery. Most importantly, as experience accumulates over time with improved endoscopic imaging and therapeutic skill levels, the clinical outcome for these patients continues to improve. While the advantages of a less invasive approach to treatment for BE neoplasia are appealing, less obvious is the anxiety to the patient that comes with the need for repeated endoscopic interventions compared with surgery. Surgical resection remains a viable option for those with disease extending beyond the mucosa and may also remain appropriate for those with early disease who choose not to have multiple endoscopies.
